# Comparison of the Values of Solar Cell Contact Resistivity Measured with the Transmission Line Method (TLM) and the Potential Difference (PD)

**DOI:** 10.3390/ma14195590

**Published:** 2021-09-26

**Authors:** Małgorzata Musztyfaga-Staszuk

**Affiliations:** Welding Department, Silesian University of Technology, Konarskiego 18A, 44-100 Gliwice, Poland; malgorzata.musztyfaga@polsl.pl

**Keywords:** transmission line model (TLM) method, potential difference (PD) method, contact resistance, resistivity, silicon solar cells, I–V characteristics

## Abstract

This work presents a comparison of values of the contact resistivity of silicon solar cells obtained using the following methods: the transmission line model method (TLM) and the potential difference method (PD). Investigations were performed with two independent scientific units. The samples were manufactured with silver front electrodes. The co-firing process was performed in an infrared belt furnace in a temperature range of 840 to 960 °C. The electrical properties of a batch of solar cells fabricated in two cycles were investigated. This work focuses on the different metallisation temperatures of co-firing solar cells and measurements were carried out using the methods mentioned. In the TLM and PD methods, the same calculation formulae were used. Moreover, solar cell parameters measured with these methods had the same, similar, or sometimes different but strongly correlated values. Based on an analysis of the selected databases, this article diagnoses the recent and current state of knowledge regarding the employment of the TLM and PD methods and the available hardware base. These methods are of interest to various research centres, groups of specialists dealing with the optimisation of the electrical properties of silicon photovoltaic cells, and designers of measuring instruments.

## 1. TLM and PD Methods—Presentation of Statistical Data Based on Electronic Databases

Information on the use of TLM transmission lines in electronics is collected in the IEEE Explore database by IEEE (Institute of Electrical and Electronics Engineers) and IET (Institution of Engineering and Technology). The method was originally proposed by Shockley in 1964 [1] and later modified by Berger [2]. Based on the data contained in the database, it can be concluded that in the period from 1990 to 2020, 17 related articles were published in journals from the Philadelphia list, including the *Journal of Photovoltaics*, *Electron Device Letters*, *Transactions on Electron Devices*, and the *Journal of Display Technology*.

Information on the use of the transmission line method was also analysed based on data contained in the Scopus database using the SciaVal tool. Based on studies from 100 countries from 2015 to 2020, a ranking of the scientific achievements of 35 countries was prepared in which Poland placed 14th. In the case of Poland, only the author of this publication published three works in the period of 2015–2020, so this is an interesting issue and a subject that requires further investigation.

Articles covering the application of the transmission line method in various fields of science from 2015 to 2020 were also analysed from the Scopus database, following the ASJC (All Science Journal Classification) classification of thematic areas. Based on this analysis, it was found that the most significant applicability of the TLM method was observed in the field of science—materials engineering (32%). In contrast, the value did not exceed 5% in energy, chemistry, or other fields. In the field of photovoltaics, from 1989 to 2020 the most significant number of publications on the application of the transmission line method in the Scopus database was recorded in 2019.

According to the Web of Science database, the topic of TLM covers the literature from 1973 to 2021, for a total of 12,996 publications. After limiting the search to the electronics category, we obtained 7722 scientific papers.

From 1983 to 2020, the IEEE Explore database in the field of electronics and electrics collected 34 publications; we searched these using the term “contact resistance and PD method”. However, from 1978 to 2020, 30 publications were recorded, of which four were in the field of photovoltaics. These were found using the keyword “contact resistance scanning,”. According to the search “contact resistance scanning” in the Scopus database, there were eight publications from 1979 to 2021, while in the Web of Science database, there were seven publications from 2005 to 2020.

Certain discrepancies in the scientific circulation of published research results in the form of scientific journals within the aforementioned bibliographic databases may result from the emergence of an increasing number of scientific journals which themselves fulfil the role of a sieve, separating the valuable scientific publications while omitting those of poor quality or that are non-scientific [3,4].

This work aims to test the value of contact resistivity of the front metallisation of solar cells. These cells were made only for measurement purposes. This work uses two methods, TLM and PD, to determine the same parameters in different ways and with different measuring stands, depending on the needs and expectations of the person performing the measurement.

The correct performance of this technological process requires the precise selection of parameters. The production of metallisation requires the correct execution of the front electrode of the correct size and shape and proper cavity in the semiconductor material. In the area where the front electrode connects to the semiconductor, junctions are formed, introducing additional resistances to the electrical circuit that limit the photocurrent flow. The use of electrode pastes minimises these losses and the level of emitter doping can be determined. There are also leakages of separated charge carriers in the junction area, causing a photovoltage drop in the solar cell. The power loss of a photovoltaic cell is influenced, among other things, by resistivity, which is a feature of cells that has a fundamental impact on their quality.

The determined parameters of the technological process strongly depend on the composition of the conductive paste or the metallic powder from which the paste is made. This is because the conductivity of the tested paste depends on the granulation, particle shape, and powder content of the paste. Ceramic glaze (e.g., SiO_2_) binds the particles of the base material with the silicon substrate; the rest is an organic carrier mixture that makes the paste sticky. The thickness of the applied layer on the front electrode and the morphology of the substrate affect the resistivity value obtained as a result of the measurements.

## 2. Methods Applied to Measuring Selected Parameters of the Electrical Properties of Photovoltaic Cells

Currently, research and development in electronics and photovoltaics are focused on developing and producing electrical contacts using various techniques and determining the dependence of the resistivity of these contacts between multicomponent metallic components and conductive layers. The goal is to optimise the technology of contact metallisation. The metallic element layer should meet various requirements to ensure the low resistivity in the vicinity of the metal–semiconductor junction. Of particular importance in a correctly performed technological process are the proper selection of the material (conductive coating and substrate); its thickness; its coating geometry (shape and size); the conditions of its production; the adhesion of the metallic element to the substrate; and the substrate morphology (e.g., structure and roughness).

The external operating parameters that characterise a silicon solar cell include the cell open-circuit voltage (V_oc_), short circuit current (I_sc_), fill factor (FF), maximum obtainable power (Pm), and photovoltaic conversion efficiency (E_ff_). These values depend, for example, on technological factors (the reflectance factor; cover factor; incomplete absorption factor due to limited material thickness; and the Q_i_ collection factor, taking into account that not all generated charge carriers reach the pn junction, where they can be separated) and are determined by the influence of the manufacturing process on the parameters, the material and physical properties of the individual layers, and the structural elements of a solar cell. The most important of these physical parameters include: the electromagnetic radiation reflection coefficient (R_ref_); the thickness of the base material of the cell (D_c_); the concentration of charge carriers (ni); the mobility (μ) and lifetime of charge carriers (τ); the length of the carrier diffusion path charge (L); the speed of surface recombination (SRV); the resistance (R) and resistivity (ρ) of areas and structural elements of the cell; and the types and concentration of defects and the resulting density of recombination centres (Nit) [5,6].

Basic measurements of photovoltaic cells include the current–voltage characteristics (I–V), which determine the physical parameters of the manufactured solar cell [7]. A one- or two-diode model can be matched numerically to the measured I–V characteristics. In the measuring system of the stand used for measuring the I–V characteristics of light and dark cells, four basic elements that determine the quality of the measurement can be distinguished: a light source, measuring system, table, and contact probes.

The I–V characteristics must be measured under the strictly defined conditions of a specific radiation spectrum and temperature. The standard used is the AM1.5 spectrum, which has an intensity of 1000 W/m^2^ at a cell temperature of 25 °C. The measured cell is placed on a brass table that acts as a current electrode for the back contact, while gold-plated probes provide the contact to the front electrode with telescopic pressure. The lighting elements are four independently positioned halogen lamps powered by a highly stabilised power supply with a “ramp” voltage increase (the so-called soft start). A reverse-biased reference photodetector controls the stability of the light intensity during the measurement. The measurement of the I–V characteristics of the cell consists of the simultaneous measurement of the voltage biasing the reverse link in the range of ±0.75 V and the measurement of the current, the value of which is calculated for the selected load resistance and the measured voltage drop.

**Figure 1:**
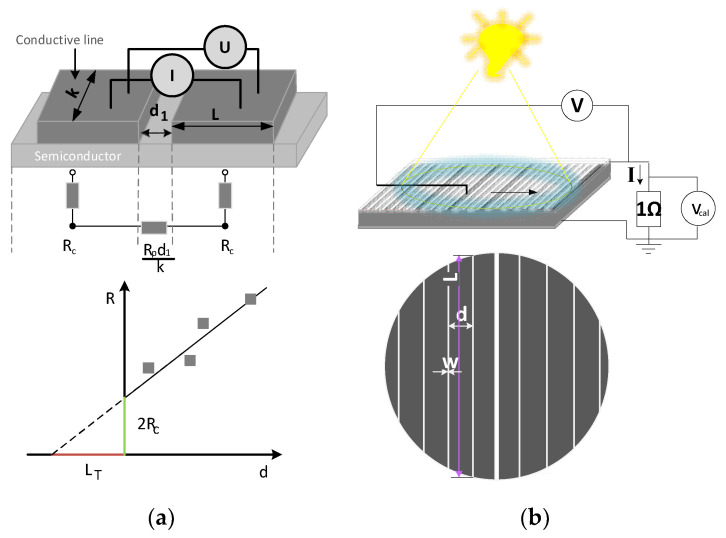
Graphical illustration of the methods of (**a**) TLM (where R—resistance; R_p_—surface resistance; I—current; U—voltage; k—electrode line length; d—distance between electrode path lines; L_T_—electrode line width to the effect of current; R_c_—contact resistance) and (**b**) PD (where w—path width; d—distance between the electrode path lines; L—path length; V—voltage on the front electrode, measured through a metal probe in direct contact with its surface) [33,34].

One of the operations used for producing photovoltaic cells is the application of electrical contacts. As numerous studies show [8,9,10,11], the electrode layer should meet various requirements to ensure the low resistance of the electrode connection zone with the substrate. Of particular importance is the appropriate selection of the material, including the electrode and substrate; the conditions of its production; the shape and size of the electrode and its adhesion to the substrate; and the substrate morphology [12,13,14]. The resistivity is understood to be the quantity that characterises the metal–semiconductor junction, considering the area above and below the junction. The value of contact resistance, which depends on the type of paste used, the substrate resistance, and the temperature of the metallisation process, can be determined experimentally—for example, by using the TLM (transmission line model) method [15,16,17,18,19,20,21,22,23,24,25] or the PD (potential differences) method [26,27,28,29,30,31,32]. In the case of the TLM method, the measurement consists of forcing an electric current signal between the selected pair of adjacent front conductive lines on the tested sample through the supply soda and the spontaneous generation of a potential difference in them through the measuring probes (Figure 1a). In the PD method, local lighting produces a current and the voltage is measured with a metal probe placed on the front metallisation of the test sample (Figure 1b). During the measurement, the sample is short-circuited with an external receiver.

**Figure 2:**
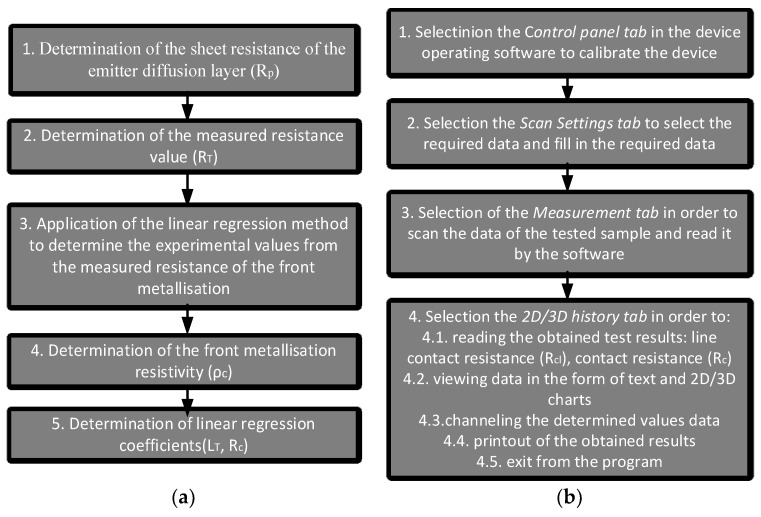
Sequence of actions when carrying out measurements using the (**a**) TLM and (**b**) PD methods.

Figure 2 shows the method for measuring using two measurement techniques [33,34].

Table 1 shows the procedure for determining the selected electrical parameters (including resistivity) of semiconductor structure contacts using the aforementioned measurement methods. In order to standardise the results obtained from the electrical properties tests, this article adopts one determination of contact resistance (R) and resistivity (ρ) for two methods.

Measurements of the resistivity of the front contacts of the semiconductor structures were carried out in two research centres and designated as the research centre A_TLM method and the research centre B_PD method. Table 2 presents the differences between the methods used and the research equipment used for this purpose.

In the case of the TLM method, the measurement is performed manually but is repeatable. The mere collection and processing of the measurements were time-consuming. The user has to develop a form for the graphical presentation of the received data.

In the PD method, the measurement is performed automatically, and the user decides how long it is to last. This stand enables the immediate recording of the measurement results in 2D and 3D formats with the calculated data and their direct printout. However, it is impossible to perform the measurement again because the tested sample is very damaged. The patterns of the applied front metallisation, sample size, cost and size of the test stand, number of patterns used to determine the parameters sought, and the symbols of these parameters (Table 1) are the main differences between these methods. The symbols of the same parameters may differ because they often depends on the manufacturers bringing something new to the market. In the case of Mechatronics, this was a stand equipped with a Corescan device.

## 3. Methodology

**Figure 3:**
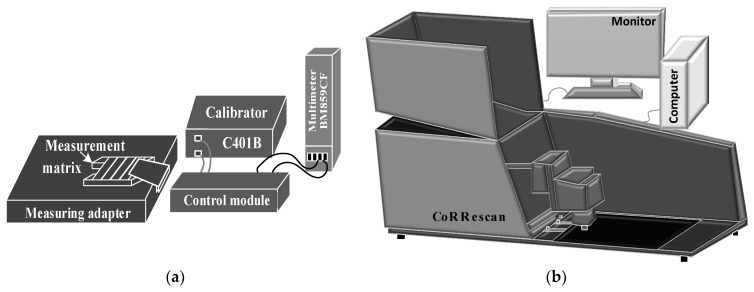
Determination of electrical parameters by the method of (**a**) TLM with the use of laboratory equipment and (**b**) PD with the use of industrial equipment.

The test apparatus with the applied method was used to test the selected electrical parameters (i.e., resistance and resistivity): (1) TLM [11] (Figure 3a) and (2) PD (Figure 3b) [26,33].

The measuring stand used for the TLM method is a measuring system developed as part of a research project [16]. The proposed solution used for measuring the selected electrical parameters with the TLM method was granted a patent by the Patent Office of the Republic of Poland in 2014—P.398223. On the other hand, the measuring stand used for the PD method was introduced to the market in 2000. It was designed by SunLab (an ECN spin-off) and manufactured and delivered to the market by Mechatronics (under license from SunLab) [26].

As part of the experiment, the selected electrical parameters (i.e., resistance and resistivity of photovoltaic cells) were tested, and then, using the formulae contained in Table 1, their values were determined [34]. In the TLM method, a row of five front electrodes (strip size—0.1 mm × 8 mm) with a variable distance between them (2.5; 5; 10; 30 mm) was used. The electrode height was 15 µm and the surface resistance of the emitter diffusion layer was 50 Ω/□. A current value of 30 mA was used for the TLM method, while for the PD method a current density value of 30 mA/cm^2^ was used. The front electrode was made of DuPont standard PV19b silver paste, which is commonly used in the industry. The front electrode was applied using a template. The tests were carried out on two series of samples, which differed in terms of the firing temperature used in the front metallisation in the belt furnace, selected to obtain the objectively most advantageous product feature—i.e., the solar cell and the measurements performed.

A total of 32 samples were used for this investigation. This paper presents the selected results of samples produced with the use of the methods described. The research was carried out in a narrow scope. For this article, samples were selected with layer parameter distributions similar to a Gaussian distribution. The limit values from the interval were given for each series, which meant the minimum value of the resistivity of the front electrode connection with the substrate.

## 4. List of Test Results Performed with the Use of the TLM and PD Methods

**Figure 4:**
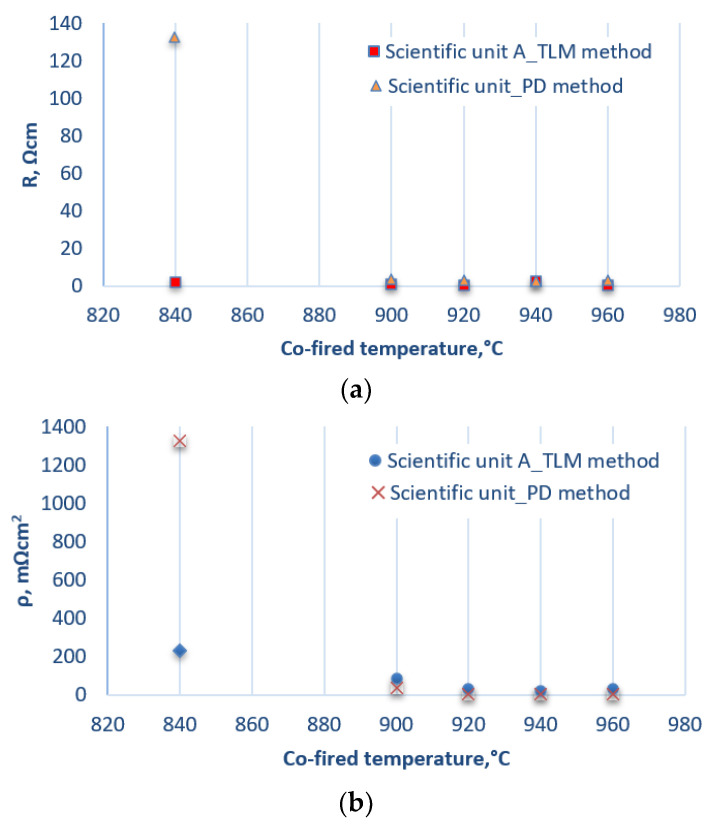
List of electrical parameters—(**a**) resistance and (**b**) resistivity—of photovoltaic cells with a front electrode applied using a template and made of commercial PV19B paste [34].

**Figure 5:**
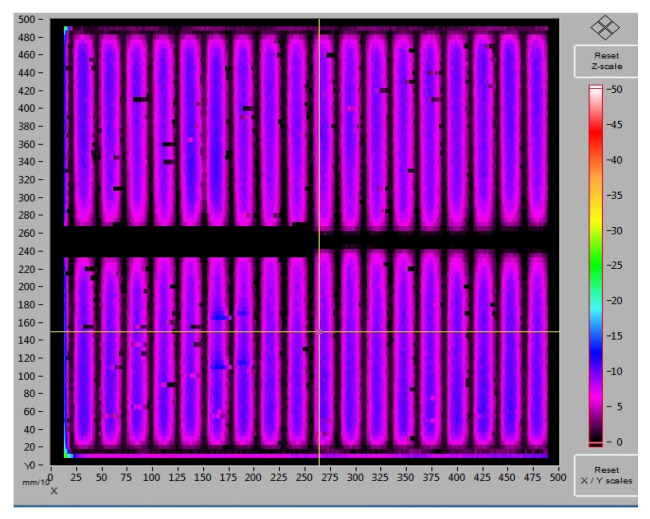
An example of the graphical distribution of the resistance of a photovoltaic cell with front metallisation fired in an infrared IR furnace at a temperature of 940 °C (original 2D printout from the Corescan device software).

Figure 4 shows the results of measurements of the electrical properties of the first series of photovoltaic cells [34]. Based on the results obtained by testing the electrical properties of this series of photovoltaic cells, it was found that the lowest values for resistance and resistivity were obtained from samples with a firing temperature of 940 °C for both methods (TLM and PD). These values were R = 0.4 Ω, ρ = 18 mΩ cm^2^ for the TLM method and R = 2.5 Ω cm, ρ = 20 mΩ cm^2^ for the PD method (Figure 5). In the case of cells where the electrodes were fired at a low temperature—e.g., 840 °C—it can be concluded that the discrepancies in the measurements of the resistance and resistivity values from both methods resulted from the poor connection of the electrode with the substrate.

**Figure 6:**
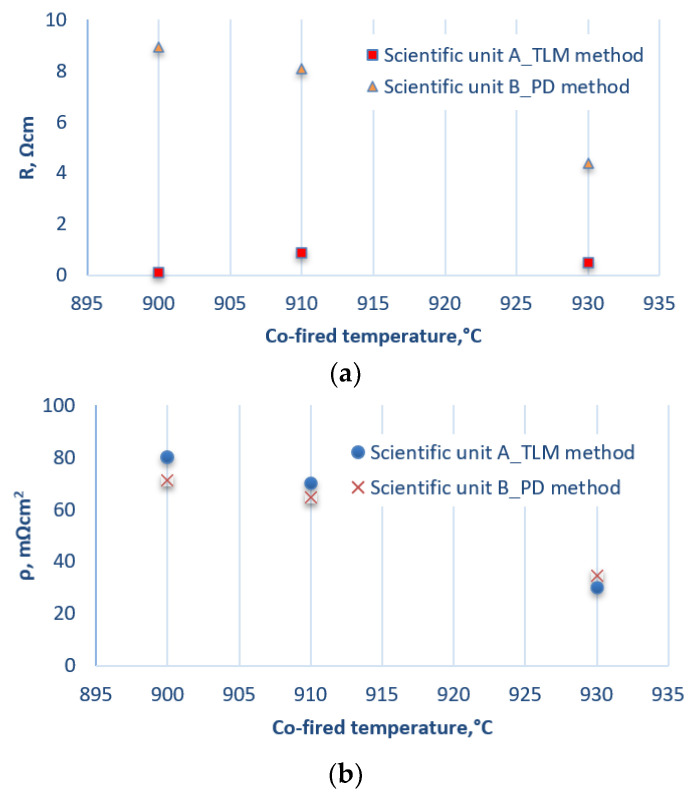
List of electrical parameters—(**a**) resistance and (**b**) resistivity—of photovoltaic cells with a front electrode applied using a template and made of commercial PV19B paste.

**Figure 7:**
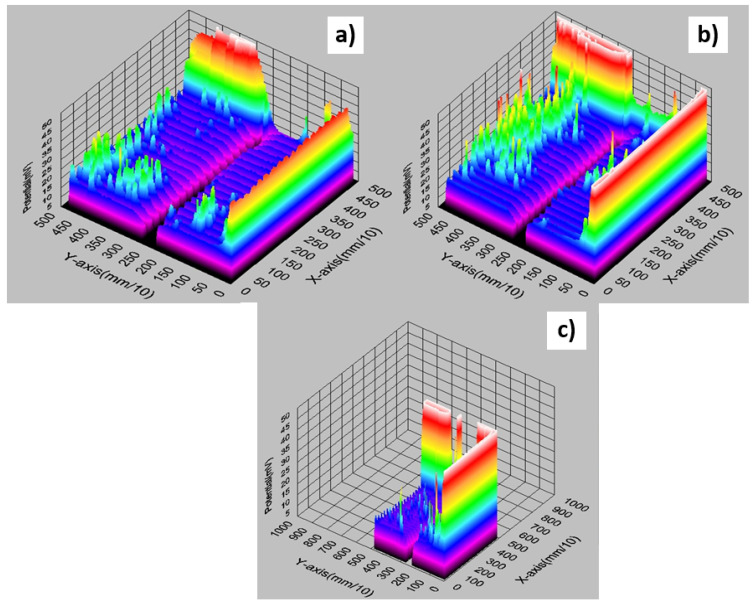
An example of the graphical distribution of the resistance of photovoltaic cells with front metallisation fired in an infrared IR furnace at different temperatures: (**a**) 900 °C, (**b**) 910 °C, (**c**) 920 °C (original 3D print from the Corescan device software).

Figure 6 shows the results of measurements of the electrical properties of the second series of photovoltaic cells. Based on the results of this series of tests, it was found that the lowest values of resistance and resistivity were obtained for samples with a firing temperature of 930 °C for both methods (TLM and PD) (Figure 6b). These values were R = 0.5 Ω, ρ = 30 mΩ cm^2^ for the TLM method and R = 4 Ωcm, ρ = 35 mΩ cm^2^ for the PD method. Figure 7 presents an original measurement printout from the Corescan device of the resistance distribution of the cells.

To summarise, based on the comparative analysis of the first series of photovoltaic cells using the TLM and PD methods, it was found that the measurements of resistivity in a temperature range of 900 to 960 °C are of the same or similar order. It can also be stated that in a temperature range of 840 to 900 °C, the cells showed the highest and most non-uniform contact resistance, which resulted in the dispersion of the obtained resistivity values. At 940 °C, the cell with the lowest contact resistance without damage was obtained.

In the second series, the results of the resistance values at the metal–semiconductor contact were scattered. Discrepancies in the obtained data may have resulted from, among other things, the manual application of the front metallisation—e.g., using a template—or another stage in the technological process of producing a finished photovoltaic cell. The results of the resistivity measurements in the accepted temperature range are of a similar order. One can also notice some defects in all samples. This is confirmed by the results of the risk at the metal–semiconductor interface. The lowest resistance value was obtained at 930 °C.

After the analysis, it can be unequivocally stated that each of the described methods can be used to collect data and information that can be used in the cell manufacturing process. The dispersion of their values may result from, among other things, the technology used in manufacturing the finished product; production automation (which reduces costs and significantly increases productivity); the robotisation of control stations; the methods and indicators used in the product quality assessment at every stage of production; and the mathematical formulae available in the literature for their calculation and analysis.

## 5. Summary

1. Both series of samples were made using the same technology. The measurement methods used in two independent research centres made it possible to compare their advantages and disadvantages. The potential difference method allows the modelling of the contact resistance of the front electrode’s frontal contact mesh and the optimisation of the burnout process. In addition, it is possible to graphically present the contact resistance measurements of the front electrode of the photovoltaic cell in 2D/3D, which is very useful for detecting possible defects at this stage of the technological process. This is not possible in the transmission line method. The PD method is destructive, while in the TLM method, depending on the measuring probes used, the measurement can be repeated. Automatic measurement and adjustment of measurement parameters is another advantage of the PD method, but the dimensions and the cost of purchasing the entire stand can be classified as disadvantages. Performing the measurements manually is associated with an extended time of implementation, so this can be considered a disadvantage of the TLM method, while the cost of purchasing the station itself is low, which is clearly an advantage.
2. The researcher/research team, depending on their requirements or the needs of the industrial market, decide on the choice of method and a suitable measuring station.
3. Based on the research analysis (Figure 4b and Figure 6b), it can be concluded that the measured values of resistivity may differ slightly from each other. This is mainly due to the formulae available in the literature, the number of measurements performed, and the technological processes used, often the lack of automatic line technology in the cell manufacturing process. The analysis mentioned above proves the credibility of the obtained results of electrical measurements using the TLM method in research centre A and the PD method in research centre B, as well as the legitimacy of their use in testing photovoltaic cells.
4. The TLM method has a few problems. Firstly, the fit of the straight line to the measurement points, expressed by the value of its slope, is subject to uncertainty. A more significant problem, however, is the correct determination of the value of the layered resistance used to calculate the resistance between the two contact lines. This is because, in the process of forming the contact between the paste and the substrate, there is a change in the layer resistance directly below the contact line. Secondly, the method does not take into account the resistance of the metal on the contact line. The advantage of the TLM method is a simple sample preparation procedure (no need to use a ready-made sample of solar cell) and quick and direct measurement of the resistance values. It is also a non-destructive method. The required components of the stand are calibrated according to the guidelines of the manufacturers of these components. The measuring station enables:
  - Setting the current value on the C401B calibrator in a range from 0 to 110 mA with a resolution of 0.01 mA, with an accuracy of ± (0.1% of the set value + 6 digits);
  - Voltage measurement with the BM859CF voltmeter in a range from 0 to 500 mV with a resolution of 0.01 mV, and in a range from 0.5 to 50 V with a resolution of 0.1 mV and an accuracy of ± (0.02% of the set value +2 digits).

In the case of the PD method, the problem is the precise determination of the current density value necessary in the measurement procedure. This can be performed using a high-class monochromator, measuring the spectral efficiency of a cell—e.g., silicon—in a range of 300–1100 nm; multiplying it by the photon flux; and then integrating it over the entire range and calculating the J_sc_ of the cell. The second method is to measure the bright I–V characteristic and calculate J_sc_, but this is also subject to uncertainty. The second disadvantage of the method is its destructive procedure that does not allow repeated measurements of the sample in the same area. This necessitates the use of samples in the form of a ready cell. The manufacturer is responsible for the calibration of the device, and the measurement accuracy refers to a constant value in the formulae (i.e., “correction factor” (* 1.8) for current leakage out of the spot and shading by the probe), which is beyond the control of the user. On the other hand, the advantage of the PD method is the ability to determine the quality of the sample (e.g., its defects) based on the graphical observation of the result and, more importantly, the determination of contact homogeneity (i.e., the low or high uniformity of the contact resistance value).

## Figures and Tables

**Table 1:** Formulae and quantities characterising the metal–semiconductor junction according to the method applied.

No	TLM Method	Literature	No	PD Method	Literature
A1.	$R_{p}=\frac{U}{I}\cdot K$, Ω/□	[17,20,21,22,23,24,25]	B1	$R_{cl}=C\cdot \frac{V}{I^{\prime }}=C\cdot \frac{V}{d\cdot J}$, Ω·cm $I^{\prime }=\frac{I}{L}=J\cdot d$, A/cm	[26,27,28,29,30,31,32,33,34]
A2.	$R_{T}=\frac{U}{I}$, Ω				
A3.	$R_{T}=2R_{c}+\frac{d\cdot R_{p}}{k}$, Ω			$R_{c}=C\cdot \frac{V}{I^{\prime \prime }}=C\cdot \frac{V\cdot w}{J\cdot d}$, Ω·cm^2^ $I^{\prime \prime }=\frac{I}{\left(L\cdot w\right)}=\frac{J\cdot d}{w}$, A/cm^2^	
A4.	$L_{T}={\left(\rho_{c}/R_{p}\right)}^{0.5}$, cm $R_{c}=\frac{\sqrt{\rho_{c}\cdot R_{p}}}{k}\coth\;\left(L\cdot L_{T}\right)$, Ω				
A5.	If, L_T_ ≥ 2 L, to $\rho_{c}=R_{c}\cdot k\cdot L_{T}$ Ω·cm^2^				
	If, L_T_ < 2 L, to $\rho_{c}=R_{c}\cdot k\cdot L$ Ω·cm^2^				

Where C—correction factor (·1.8) for the current leakage out of the spot and shading by the probe.

**Table 2:** Primary specifications of the available research equipment [26,33].

No	Feature	Type of Measuring Stand	
		Laboratory	Industrial
1.	Test sample size (thickness, length × width)	200–1000 μm, 50 mm × 50 mm	200–1000 μm, 25–215 mm × 40–215 mm
2.	Pattern of the produced front metallisation	A series of parallel track lines with varying distances between them	Busbar with collecting tracks
3.	Measurement method used	TLM	PD
4.	Measurement mode	Manual	Automatic
5.	Time consumption	Short measurement time	The optimal one depending on the operator settings in the software
6.	Measurement data output	Graphical and textual	Graphical 2D/3D and textual
7.	Printout of measurement data	No	Yes
8.	Method type	Destructive/Nondestructive *	Destructive
9.	Test device cost	Low	High
10.	Dimensions L × W × H (length, width, height)	TLM stand: 1000 mm × 1000 mm × 300 mm	Corescan system: 515 mm × 809 mm × 350 mm
11.	Measurement accuracy	(1) Digital multimetr: ±(0.02% of the set value + 2 digits) (2) Calibrator: ±(0.1% of the set value + 6 digits)	-

* depends on the application of the probes.

## References

[1] Shockley W. (1964). Research and Investigation of Inverse Epitaxial UHF Power Transistors. https://www.readcube.com/articles/10.21236%2Fad0605376.

[2] Berger H.H. Contact resistance on diffused resistors. Proceedings of the IEEE Solid-State Circuits Conference.

[3] https://depot.ceon.pl/bitstream/handle/123456789/15614/Aneta_Drabek_Indeksowanie_czasopism_w_referencyjnych_bazach_danych.pdf?sequence=1&isAllow.

[4] https://publications.jrc.ec.europa.eu/repository/bitstream/JRC123157/jrc123157_online_2.pdf.

[5] Panek P. (2011). Polish Photovoltaics 2011.

[6] Bayod-Rújula A.A. (2019). Solar Photovoltaics (PV).

[7] Jordan D.C., Kurtz S.R. (2012). Photovoltaic Degradation Rates—An Analytical Review. Prog. Photovolt. Res. Appl..

[8] Enebish N., Agchbayar D., Dorjkhand S., Baatar D., Ylemj I. (1993). Numerical Analysis of Solar Cell Current-Voltage Characteristics. Sol. Energy Mater. Sol. Cells.

[9] Rodacki T., Kandyba A. (2000). The Energy Processing in Solar Station.

[10] Green M.A. (1986). Technology and System Applications: Solar Cells. Operating Principles.

[11] Pysch D., Mette A., Filipovic A., Glunz S.W.A. (2009). Comprehensive Analysis of advanced solar cell contacts consisting of printed fine-line seed layers thickened by silver plating. Prog. Photovolt. Res. Appl..

[12] Schroder D.K., Meier D.L. (1984). Solar cell contact resistance—A review. IEEE Trans. Electron Devices.

[13] Urban T., Heitmann J., Müller M. (2020). Numerical Simulations for In-Depth Analysis of Transmission Line Method Measurements for Photovoltaic Applications—The Influence of the p–n Junction. Phys. Status Solidi A.

[14] Scharlack R.S. (1979). The Optimal Design of Solar Cell Grid Lines. Sol. Energy.

[15] Szlufcik J. (1989). Study of the Conditions of Using Thick-Film Technology for the Production of Solar Cells from Monocrystalline Silicon. Ph.D. Thesis.

[16] Musztyfaga-Staszuk M. (2011). Laser Micromachining of Silicon Photovoltaic Cells. Ph.D. Thesis.

[17] Dobrzański L.A., Musztyfaga M., Drygała A., Panek P. (2010). Investigation of the screen printed contacts of silicon solar cells from Transmissions Line Model. J. Achiev. Mater. Manuf. Eng..

[18] Woelk E.G., Kräutle H., Beneking H. (1986). Measurement of low resistive ohmic contacts on semiconductors. IEEE Trans. Electron Devices.

[19] http://tuttle.merc.iastate.edu/ee432/topics/metals/tlm_measurements.pdf.

[20] Schroder D.K. (2006). Semiconductor Material and Device Characterisation.

[21] Grover S., Sahu S., Zhang P., Davis K.O., Kurinec S.K. Standardisation of Specific Contact Resistivity Measurements using Transmission Line Model (TLM). Proceedings of the 33rd IEEE International Conference on Microelectronic Test Structures.

[22] Gregory G., Li M., Gabor A., Anselmo A., Yang Z., Ali H., Iqbal N., Davis K. (2019). Nondestructive Contact Resistivity Measurements on Solar Cells Using the Circular Transmission Line Method. J. Photovolt..

[23] Hocine R., Belkacemi K., Kheris D. 3D-analytical method analysis of thermal effect in space shaded solar panel. Proceedings of the 9th International Conference on Recent Advances in Space Technologies, RAST.

[24] Mir H., Arya V., Höffler H., Brand A. (2019). A Novel TLM Analysis for Solar Cells. J. Photovolt..

[25] Takaloo A.V., Joo S.K., Es F., Turan R., Lee D.W. (2018). A Study on Characterisation of Light-Induced Electroless Plated Ni Seed Layer and Silicide Formation for Solar Cell Application. J. Korean Phys. Soc..

[26] https://www.energy-xprt.com/downloads/sunlab-model-corescan-mapping-of-metal-grid-contact-resistance-datasheet-736296.

[27] Musztyfaga M., Dobrzański L.A., Rusz S., Staszuk M. (2014). Application examples for the different measurement modes of electrical properties of the solar cells. Arch. Metall. Mater..

[28] Van der Heide A.S.H., Bultman J.H., Hoornstra J., Schonecker A., Wyers G.P., Sinke W.C. Optimising the front side metallisation process using the Corescan. Proceedings of the 29th IEEE Photovoltaic Specialists Conference.

[29] Van der Heide A.S.H., Goris M.J.A.A. Contact optimisation on lowly doped emitters using the corescan on non-uniform emitter cells, Nineteenth European Photovoltaic Solar Energy Conference. Proceedings of the International Conference.

[30] Van der Heide A.S.H. Dutch Patent NL1013204, Applied 4 October 1999, Granted 5 April 2001, Worldwide Patent Pending. https://patentimages.storage.googleapis.com/bf/9e/60/5bd068bd0580b6/NL1013204C2.pdf.

[31] Van der Heide A.S.H., Bultman J.H., Hoornstra J., Schönecker A., Wyers G.P., Sinke W.C. Locating losses due to contact resistance, shunts and recombination by potential mapping with the Corescan. Proceedings of the 12th Workshop on Crystalline Silicon Solar Cell Materials and Proceses.

[32] Van der Heide A.S.H., Schönecker A., Wyers G.P., Sinke W.C. Mapping of Contact Resistance and Locating Shunts on Solar Cells Using Resistance Analysis by Mapping of Potential (RAMP) Techniques. Proceedings of the 16th European Photovoltaic Solar Energy Conference.

[33] Musztyfaga-Staszuk M. (2019). New Copper-Based Composited in Use for Fabrication of the Silicon Photovoltaic Cells.

[34] Musztyfaga-Staszuk M., Janicki D., Panek P. (2019). Correlation of different electrical parameters of solar cells with silver front electrodes. Materials.

